# Apical Hypertrophic Cardiomyopathy Among Non-Asians: A Case Series and Review of the Literature

**DOI:** 10.14740/cr459w

**Published:** 2016-02-20

**Authors:** Karan Kapoor, Amal Chaudhry, Matthew C. Evans, Amish Sura

**Affiliations:** a University of Maryland Medical Center, Baltimore, MD, USA; b Mercy Medical Center, Baltimore, MD, USA

**Keywords:** Hypertrophic cardiomyopathy, Apical variant, Echocardiography

## Abstract

Apical hypertrophic cardiomyopathy (AHCM) has been rarely described in the Western world. More recently, improved sensitivity of diagnostic modalities and increased diagnostic awareness have increased detection rates, suggesting that the prevalence outside of Asia may have been previously understated. Hallmark features of AHCM include deeply negative, “giant” T-wave inversions on electrocardiography and a “spade-like” configuration of the left ventricle on ventriculography. We present two cases of AHCM, one in an African-American female and another in a Caucasian male.

## Introduction

Apical hypertrophic cardiomyopathy (AHCM) is found in up to 25% of Japanese patients with hypertrophic cardiomyopathy (HCM), but outside of this population, it represents a markedly uncommon morphologic variant of HCM, with most reports suggesting a prevalence between 1% and 3% [1-3]. The basis for differences in the phenotypic expression of apical hypertrophy between Asians and non-Asians has not been elucidated. Although most patients with AHCM experience minimal to no symptoms, presentations with a variety of signs and symptoms including atrial fibrillation, ventricular tachycardia and angina have been described [1]. Typical features of AHCM include T-wave inversion, particularly in the left precordial leads of the electrocardiogram, and a “spade-like” configuration of the left ventricular cavity at end-diastole on left ventriculography [4, 5]. Cardiac catheterization (and subsequent ventriculography) or cardiac magnetic resonance imaging (cMRI) is often needed to establish the diagnosis, which can be missed on two-dimensional transthoracic echocardiography. Advances in non-invasive imaging techniques, and in particular, cMRI, have led to its establishment as the gold standard diagnostic modality given the invasive nature of cardiac catheterization [6, 7]. Although generally associated with a better prognosis than other forms of HCM, serious cardiac complications have been described, including progressive heart failure, myocardial infarction and sudden-cardiac death [1, 8]. We describe two cases of AHCM in non-Asian patients.

## Case Reports

### Case 1

**Figure 1:**
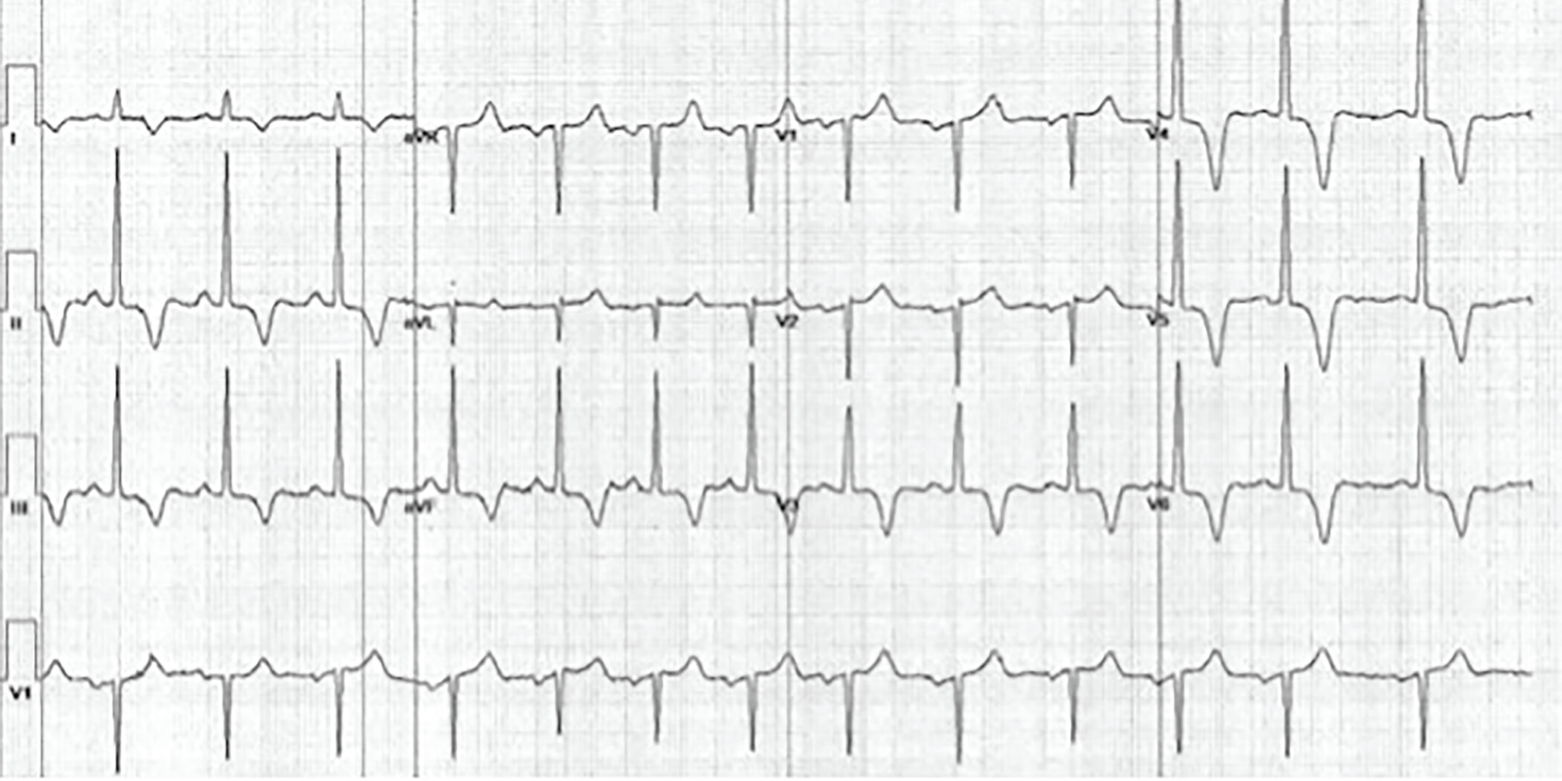
Resting EKG of the patient from case 1. EKG illustrates marked T-wave inversions in leads II, III, aVF, and V3-V6, a typical feature of AHCM.

A 28-year-old African-American woman presented with a several hour history of typical angina. She provided a history of hypertension, hyperlipidemia, metabolic syndrome, and a family history of sudden cardiac death in her 23-year-old brother. Associated symptoms included palpitations, progressive orthopnea, and pedal edema. Vital signs were all within normal limits. Cardiac examination disclosed a prominent S4. Electrocardiography (EKG) revealed left ventricular hypertrophy and deep T-wave inversions in leads V3-V6, but no ST-segment abnormalities (Fig. 1). Cardiac biomarkers and basic laboratory investigations were normal.

Transthoracic echocardiography revealed normal left ventricular size and function (ejection fraction 60%), and moderate-to-severe left ventricular hypertrophy. Although the apex was not well visualized, obstructive physiology was noted despite lack of a well-defined gradient.

**Figure 2:**
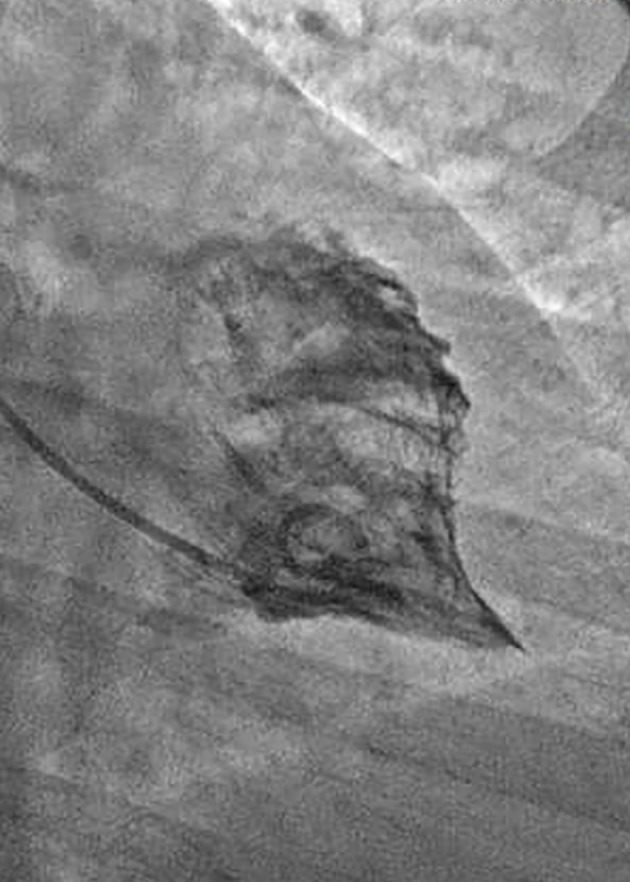
Ventriculogram from case 1 demonstrating complete obliteration of the apex in a “spade-like” or “bird’s beak” fashion. Elevation of the left ventricular end-diastolic pressure was also noted.

**Figure 3:**
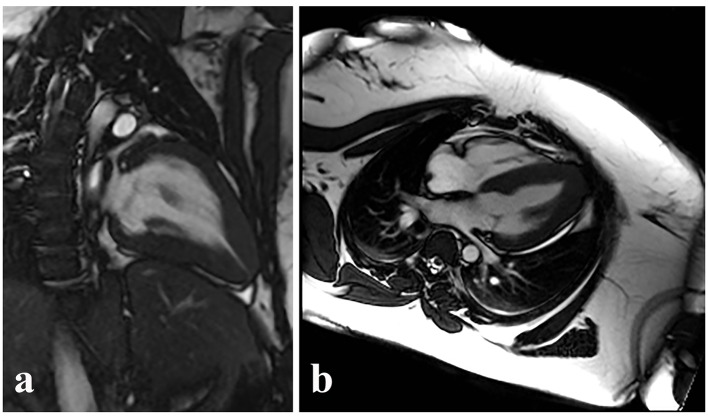
Similar ventricular morphology demonstrated on cMRI in the sagittal (a) and axial (b) planes. Video clip of systole captured during cMRI from case 1, further demonstrating the hallmark morphology of AHCM.

Coronary angiography revealed no significant epicardial coronary disease. Obliteration of the apex in a “spade-like” fashion and elevation of the left ventricular end-diastolic pressure were noted on ventriculography (Fig. 2). The diagnosis of AHCM was confirmed by cMRI (Fig. 3a, b; Supplementary video 1, www.cardiologyres.org). Given the family history of sudden cardiac death, she underwent implantation of a single-chamber internal cardioverter defibrillator.

### Case 2

**Figure 4:**
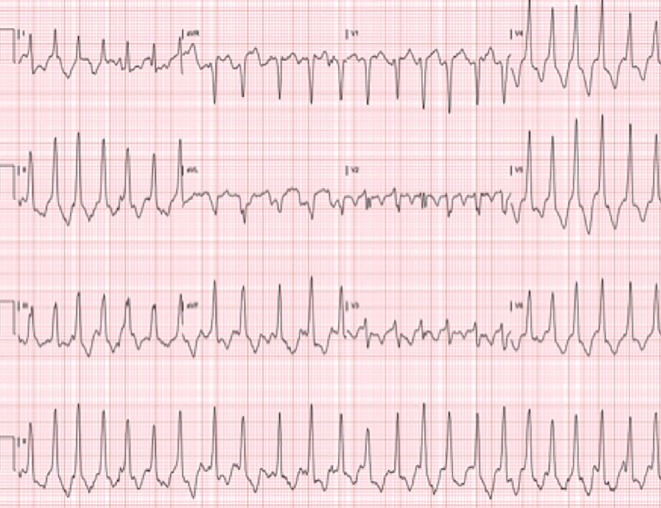
EKG from stage IV of an exercise stress test from case 2. EKG illustrates more pronounced T-wave inversion when compared to the patient’s resting EKG (not shown).

**Figure 5:**
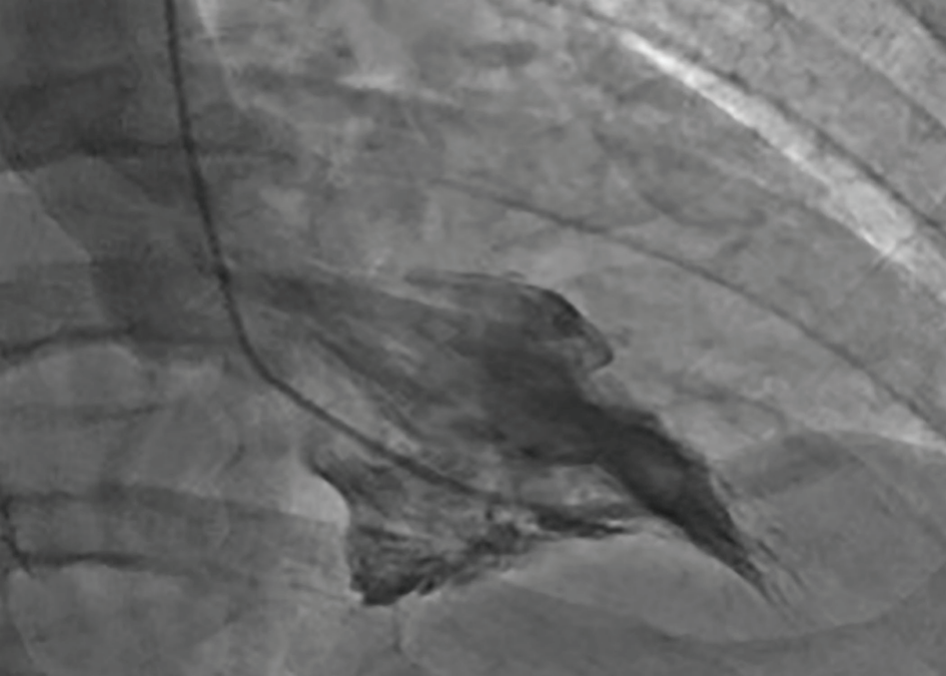
Cardiac catheterization from case 2. Ventriculogram performed during cardiac catheterization reveals “spade-like” configuration of the left ventricle, characteristic of AHCM.

A 50-year-old Caucasian man with a history of paroxysmal atrial fibrillation, obstructive sleep apnea and a family history of sudden cardiac death in two first-degree relatives presented to the outpatient clinic with palpitations. His initial diagnosis of atrial fibrillation was made 10 years prior and was attributed to excessive caffeine consumption, given that he regained a normal sinus rhythm following cessation of caffeine. At the time of atrial fibrillation diagnosis, transthoracic echocardiogram demonstrated evidence of mild left ventricular hypertrophy. Ten years later, his resting electrocardiogram demonstrated recurrence of rate-controlled atrial fibrillation, left ventricular hypertrophy and 2.5 mm deep T-wave inversion in leads V4-V6. He was placed on an event monitor and was noted to experience 22 beats of non-sustained ventricular tachycardia in addition to periods of atrial fibrillation. He underwent exercise nuclear stress testing according to a standard Bruce protocol, during which he completed 18 s of stage IV prior to experiencing shortness of breath at 100% of his maximal predicted heart rate and concomitant worsening in the degree of T-wave inversions in the inferior and lateral leads (Fig. 4). No evidence of reversible ischemia was noted. He subsequently underwent cardiac catheterization, which showed no evidence of obstructive coronary disease, but a “spade-like” configuration of the left ventricle similar to that seen in case 1 (Fig. 5). A diagnosis of AHCM was made. Given his family history and the occurrence of ventricular tachycardia, implantable cardiac defibrillator therapy was offered.

Both of our patients and their first-degree family members declined any genetic testing. At subsequent 6-month follow-up visits, both patients were asymptomatic and reported adherence to beta-blocker therapy. No inadvertent defibrillator shocks were reported.

## Discussion

The apical variant of HCM in which left ventricular wall thickening is confined to the most distal region of the apex has been regarded as a phenotypic expression of non-obstructive HCM largely limited to Japanese patients. Nevertheless, since its original description in the 1970s [4, 5], several studies have been published outside of Asia regarding this entity, albeit insufficiently powered for robust conclusions regarding natural history, prognosis or long-term management strategies [2, 8, 9]. However, the implication of these studies has been that AHCM among non-Asian patients may represent an entirely different entity that carries a different prognosis compared to the Japanese variant.

Our first case highlights the potential for AHCM to manifest early in adulthood, similar to other more commonly seen variants of HCM [10]. Most other case series involving at least 10 or more patients with the apical variant report a mean age of at least 41 years [11]. Furthermore, this case highlights the potential for AHCM to present with angina, which is followed by atypical chest pain (14%), palpitations (10%), dyspnea (6%) and pre-syncope/syncope (6%) in the minority of symptomatic individuals according to one of the larger published series [8]. Notably, Smith et al described an initial presentation of AHCM similar to case 1 (i.e. severe angina) in a 39-year-old African-American man, suggesting a potential phenotypic proclivity among young African-American patients [12].

The second case in our series is representative of the more common, older-onset AHCM [11]. Our patient had antecedent echocardiographic evidence of left ventricular hypertrophy in addition to atrial fibrillation several years prior to his declarative presentation. Delayed penetrance and late appearing left ventricular hypertrophy have been described among adults diagnosed with AHCM. More frequent surveillance imaging in the 10-year interval between his initial diagnosis of atrial fibrillation and development of palpitations may have yielded an earlier diagnosis. Interestingly, our patient fits the profile of Caucasian AHCM patients described in an Australian series of 23 patients, regarding his late and relatively abrupt onset of symptomatic AHCM [13]. Both atrial fibrillation and coronary fistulae were noted with increased frequency among these patients, although the latter was absent in our patient. The development of AHCM in older individuals may represent an example of gene-environment interaction required for phenotypic manifestations, although the exact environmental triggers are unclear [13]. Additionally, the absence of marked T-wave inversions on his resting EKG reflects prior studies suggesting that the incidence of this electrocardiographic finding diminishes considerably with advancing age [8]. Finally, pertinent to both of our cases is the fact that female gender and atrial fibrillation have been identified as predictors of mortality in retrospective studies [11].

Ethnically, AHCM accounts for 13-41% of all variants of HCM among Asian individuals, whereas the prevalence among non-Asians is < 5% [14]. However, much of these epidemiologic data are derived from studies conducted in the early 1990s, and the exact prevalence of AHCM in non-Asians may be underestimated due to diagnostic unawareness as well as the heterogeneous appearance on transthoracic echocardiogram, the most frequently utilized diagnostic modality [15]. Contrary to our two cases, previous studies have demonstrated less hypertrophy confined predominantly to the apex and T-wave abnormalities on EKG among North-American AHCM subgroups [2]. Despite the higher prevalence of AHCM among Japanese patients, clinical presentation and long-term cardiovascular outcomes appear to be similar [16]. Specifically, long-term follow up studies have shown co-morbid atrial fibrillation, apical myocardial infarction, ventricular arrhythmia and apical thrombosis with subsequent embolization to occur in up 33% of all patients irrespective of ethnicity [17]. The presence of symptoms in our two cases, severe angina in the first and symptomatic palpitations in the second, bolsters the observations of other investigators that the clinical expression of AHCM is highly variable, and likely subject to a multitude of genetic and environmental influences.

No definitive guidelines delineate the role for defibrillator implantation in AHCM patients with family histories of sudden cardiac death. Not surprisingly, expert consensus supports the use of implantable defibrillator for primary prevention in select high risk patients, notably those with one of the five following risk factors: a family history of sudden cardiac death, syncope, asymptomatic non-sustained ventricular tachycardia, an abnormal blood pressure response to exercise and a left ventricular wall thickness > 30 mm [18]. Genetic testing has also been offered to both of our patients and their first-degree family members; although contrary to other subtypes of HCM, the apical variant has only occasionally been described as a familial disease manifesting an autosomal dominant inheritance. A very limited number of sarcomere gene defects, and in particular, cardiac actin Glu101Lys, have been shown to consistently produce the AHCM phenotype [19]. The widespread availability of genetic testing has led to increased recognition of genotype-positive/phenotype-negative patients. Although at present, it is not possible to predict clinical outcomes based on the presence of individual mutations, guidelines do suggest extension of surveillance with cardiac imaging at least through mid-life (40 years of age, and beyond, in select circumstances) to detect development of overt disease [20].
